# A case of von Hippel-Lindau disease with exudative maculopathy

**DOI:** 10.4103/0974-620X.53038

**Published:** 2009

**Authors:** Basel T. Ba′arah

**Affiliations:** Vitreoretinal Division, Department of Ophthalmology, Al-Hussein Hospital, King Hussein Medical Center, Amman, Jordan

**Keywords:** Argon laser, cryotherapy, hemangioblastoma, von Hippel-Lindau disease

## Abstract

Von Hippel-Lindau (VHL) disease is a rare multisystem familial tumor syndrome of autosomal dominant inheritance. Hallmark lesions include retinal, cerebellum and spinal cord hemangioblastomas, renal cell carcinomas, adrenal pheochromocytomas, angiomatous or cystic lesions of the kidneys, pancreas, and epididymis. We report a case of VHL disease in a 26-year-old patient who presented with exudative macular edema. Ocular and systemic studies revealed the presence of retinal and central nervous system hemangioblastomas, adrenal pheochromocytoma, multiple pancreatic, and kidney cysts. The retinal angiomas were successfully treated with argon laser photocoagulation and cryotherapy.

## Introduction

Von Hippel-Lindau disease (VHL) is a multisystem familial tumor syndrome predisposing to various benign or malignant tumors: central nervous system (CNS) and retinal hemangioblastomas (RHB), renal cell carcinoma (RCC) and cysts, pancreatic tumors and cysts, pheochromocytoma, and endolymphatic sac tumors. [1–3] RHB occurs in 43% to 67% of patients with VHL.[4–6] They can be asymptomatic for years and may even regress spontaneously, but usually they grow and cause visual impairment.[4,7] One of the RHB complications is exudative maculopathy. Dollfus H,[8] reported its presence in 15% of VHL disease cases. In this report, we present a case of unilateral decrease of vision due to macular exudation as the initial manifestation of VHL disease, and summarize the ocular and systemic clinical features, along with treatment measures.

## Case Report

**Figure 1:**
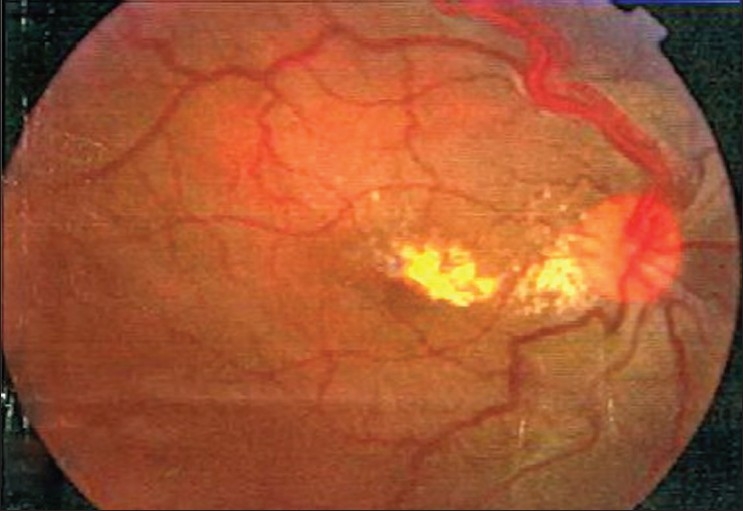
Fundus photograph of right eye, showing abundant exudates in the macular area. There is discrete enlargement of the superior temporal artery and vein

**Figure 2:**
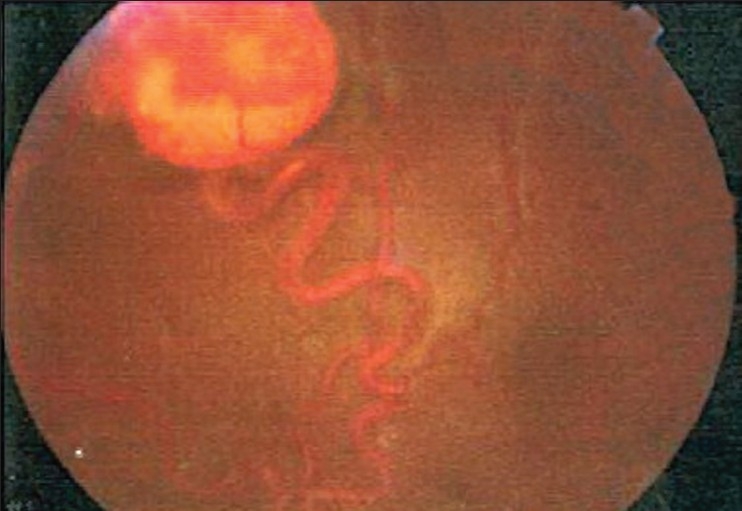
Fundus photograph of right eye. In the temporal superior periphery a retinal hemangioblastoma is present. The feeder artery and draining vein are enlarged and tortuous

**Figure 3:**
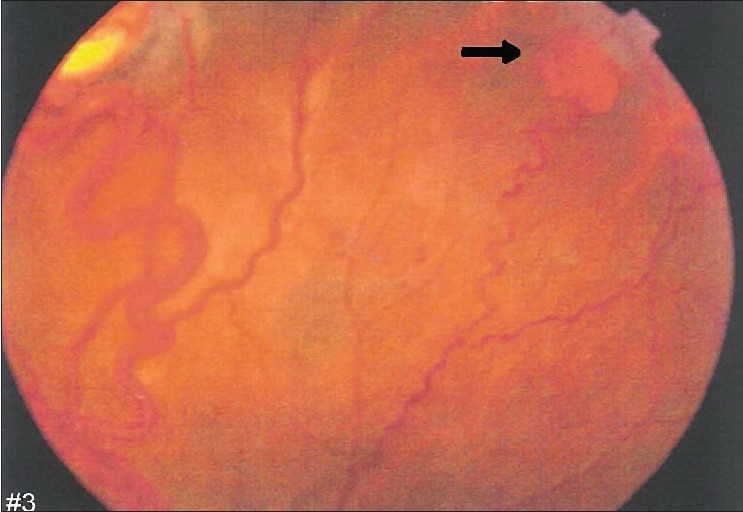
Fundus photograph of right eye peripheral retina. A small retinal hemangioma (arrow) is seen beside the large one with a moderately enlarged and tortuous draining vein

**Figure 4:**
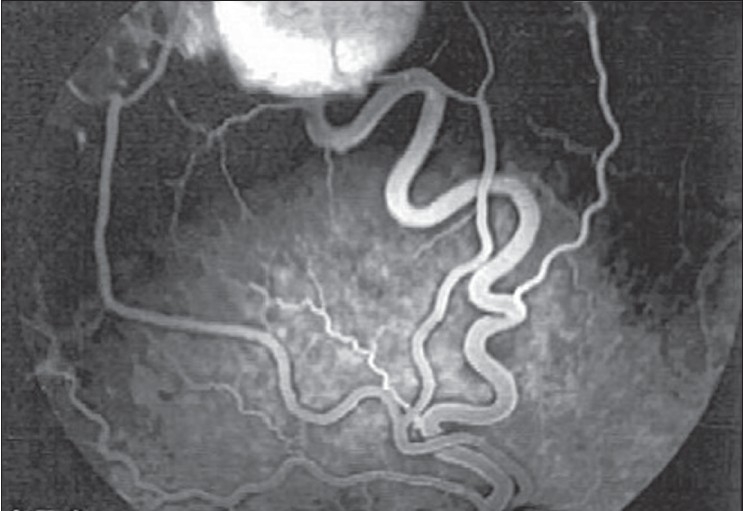
Fluorescein angiogram of right eye, showing the temporal superior retinal hemangioblastoma with the feeder artery and draining vein

**Figure 5:**
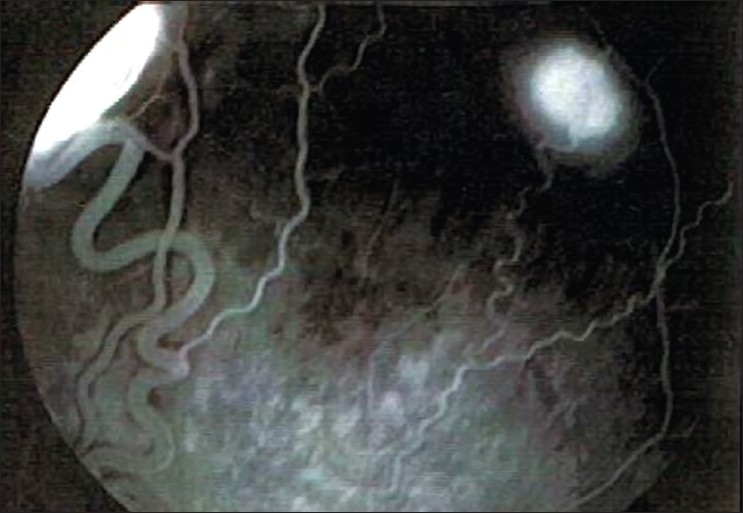
Fluorescein angiogram of right eye, showing the peripheral retinal hemagiomas

A 26-year-old man presented with painless decrease of vision in the right eye. His best corrected visual acuity was 20/200 in the right eye and 20/20 in the left. The intraocular pressure was within normal limits. The results of bilateral biomicroscopic examination of anterior segments were unremarkable. Right eye fundus examination with 78-diopter noncontact lens revealed significant macular edema with lipid exudates [Figure 1]. In addition, two lesions in the superior retina were noticed; one large (3.0 mm) with a globular reddish appearance, dilated feeding vessel and tortuous draining vein [Figure 2]; and another small (1.2 mm) with a moderately enlarged and tortuous draining vessel, which is a characteristic for RHB []Figure 3]. Two small peripheral RHBs were seen in the left eye. Fluorescien angiography showed a progressive hyperfluorescence with late leakage of fluorescien into surrounding retina [Figures 4 and 5]. Systemic studies, including magnetic resonance imaging of the brain, renal ultrasonography, and computed tomography of the abdomen revealed brain stem tumor invading medulla oblongata, right adrenal gland tumor (pheochromocytoma) and multiple pancreatic and kidney cysts.

Detailed family history revealed that his father had died from cerebral vascular accident at the age of 54; he was treated for uncontrolled blood pressure, but was not investigated for possible presence of adrenal gland tumor.

On the basis of clinical and investigational findings, the diagnosis of VHL disease was made.

**Figure 6:**
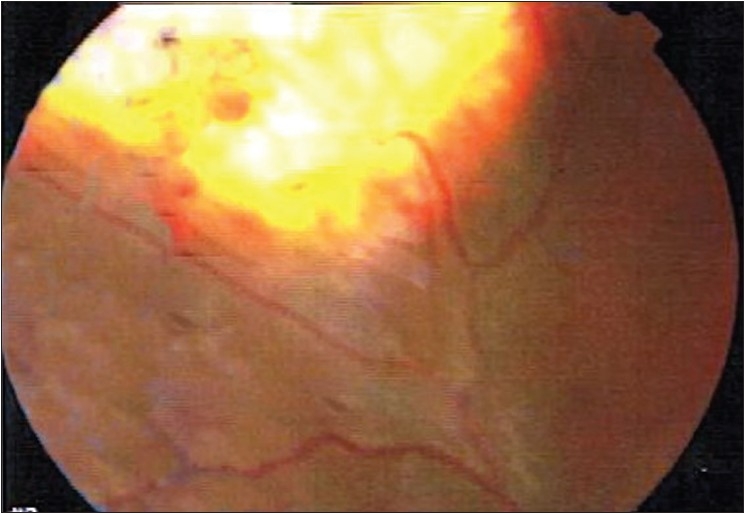
Fundus view after six months of laser photocoagulation and cryotherapy. There is complete regression of the large retinal hemangioma with formation of a choroidoretinal scar

**Figure 7:**
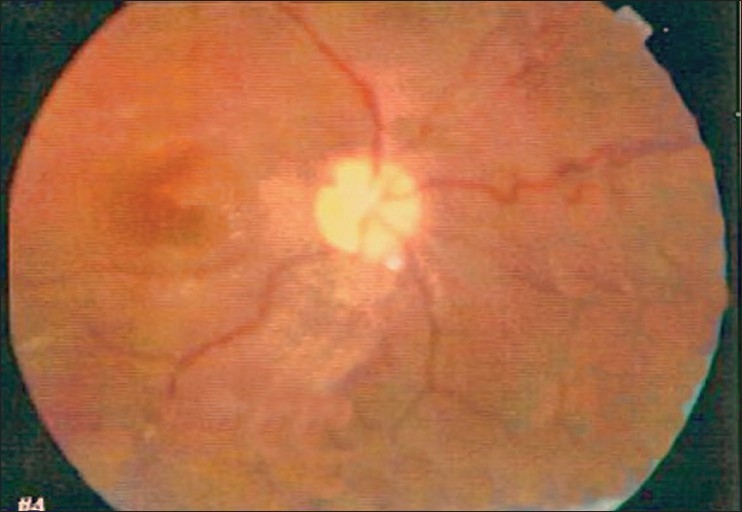
Fundus photograph of the right eye at six months after the treatment showing macular edema regression

Argon laser photocoagulation was used to treat the small (≤1.5 mm) hemangiomas. Large size (500 micron), low intensity (250 mW), and long duration (0.5 sec) laser burns were applied directly on the RHB. As for the large hemangioma, a combination therapy of laser photocoagulation and cryopexy was used. Laser burns were directly applied on the RHB and the feeding artery. This resulted in complete regression of RHBs with formation of chorioretinal scar [Figure 6]. After six months, the macular edema with exudates regressed and the visual acuity improved to 20/40 in the right eye [Figure 7]. During the next two years follow-up time, no new RHB were observed. In the third year of observation, the patient underwent right adrenalectomy and he died the day after surgery from uncontrolled internal bleeding.

## Discussion

The incidence of VHL disease in northwest England is 1 in 45,000 live births.[9] The disease is a dominantly inherited familial cancer syndrome that predisposes the patient to the development of specific tumors both benign and malignant.[10] The diagnosis of VHL disease is based on family history and/or clinical presentation of hemangioma, renal cell carcinoma, pheochromocytoma, pancreatic, and visceral cysts.[11] After identification of the VHL tumor-suppressor gene on the short arm of chromosome 3 (3p25-26); the diagnosis can be supported by molecular genetic analysis.[12] RHBs are the first manifestation in approximately half of the VHL disease patients and is usually bilateral and multifocal or becomes so over the years.[13,14] In spite of its benign nature and slow-growing course, RHB may cause sight-threatening complications such as macular exudation, retinal traction, retinal detachment, neovascular glaucoma, vitreous hemorrhage, and phthisis.[15] RHB are more commonly found in the peripheral retina.[16] The differential diagnosis of RHB includes Coat′s disease, racemose hemangioma, retinal cavernous hemangioma, retinal macroaneurysm, and vasoproliferative tumor. As a diagnostic technique, fluorescein angiography plays a major role in detecting, localizing, and observing small RHBs. The visual prognosis is frequently poor in patients with VHL disease; early detection and treatment can change the visual prognosis.[17,18] That was the case with our patient, where we started the treatment as soon as VHL disease was diagnosed. Accordingly, exudative maculopathy regressed and the vision improved. The treatment decisions are based on size and location of RHB. Careful observation is suggested for juxtapapillary and peripheral lesions less than 500 micrometer in diameter that are not associated with exudation or subretinal fluid and are not visually threatening.[19,20] Lesions of less than 4.5 mm on diameter could be treated with argon laser, but when the lesion is more than 3.0 mm in diameter it is preferable to do cryotherapy.[21] In our case, we preferred to use both techniques in ablating the large (3.0 mm) RHB; the techniques proved to be effective in destructing it.

Other possible treatment modalities include plaque radio therapy, antiVEGF therapy, photodynamic therapy, transpupillary thermotherapy, and parsplana vitrectomy.[22–24]

Most commonly, the death of VHL disease patients is related to complications of cerebellar hemangioma and metastatic renal cell carcinoma.[25,26,1] Our patient died from complications related to the surgical intervention on adrenal gland tumor (pheochromocytoma) to control blood pressure.

In summary, VHL disease is an inherited and life-long disease. Family members of the patient should undergo clinical and/or molecular genetic screening while the patient should undergo annual clinical screening after ablating the RHBs. Macular edema with lipid exudates is usually an early complication of RHB. Prompt diagnosis and ablative treatment with laser and cryotherapy appears to be effective in controlling VHL disease progression in the retina and gives a favorable visual prognosis.
